# Transport mechanical ventilation during cardiopulmonary resuscitation in out-of-hospital cardiac arrest: an exploratory prehospital pilot study

**DOI:** 10.1186/s12873-026-01692-8

**Published:** 2026-07-29

**Authors:** Welawat Tienpratarn, Pariyakon Kusolthammarat, Chaiyaporn Yuksen, Suraphong Phongsawad

**Affiliations:** 1 Department of Emergency Medicine, Faculty of Medicine Ramathibodi Hospital, Mahidol University, 270 Rama VI Road, Thung Phaya Thai, Ratchathewi, Bangkok, 10400 Thailand; 2 Department of Emergency Medicine, Faculty of Medicine Ramathibodi Hospital, Chakri Naruebodindra Medical Institute, Mahidol University, Samut Prakan, Thailand

**Keywords:** Mechanical ventilation, Bag-valve ventilation, Out-of-hospital cardiac arrest

## Abstract

**Background:**

Evidence regarding the use of mechanical ventilation (MV) during cardiopulmonary resuscitation (CPR) in out-of-hospital cardiac arrest (OHCA) remains limited, particularly in prehospital settings.

**Objective:**

To evaluate the feasibility and generate preliminary physiologic data regarding transport MV compared with bag-valve ventilation (BV) during CPR in OHCA.

**Methods:**

This prospective, time-sequenced, exploratory pilot study, conducted between September 2024 and October 2025, included adult OHCA patients aged ≥ 18 years managed by the Ramathibodi Emergency Medical Operation team. Patients received BV during the first 6 months, followed by MV thereafter, delivered using a HAMILTON-T1 transport mechanical ventilator in volume-controlled mode (tidal volume: 6–8 mL/kg; respiratory rate: 10 breaths/min; PEEP: 0 cmH₂O; I:E ratio: 1:5; FiO₂: 1.0; flow trigger: off). Ventilation was assessed using arterial or venous PCO₂ obtained after ≥ 3 min following intubation or upon return of spontaneous circulation (ROSC). As a pilot study, this investigation was not powered to detect differences in clinical outcomes.

**Results:**

Forty-one patients were analysed (29 BV, 12 MV). The mean age was 67 ± 15 years in the BV group and 66 ± 20 years in the MV group, with 55.2% and 66.7% male patients, respectively. No statistically significant differences were detected in PCO₂, pH, or pO₂ between groups. Clinical outcomes, including sustained ROSC and 28-day survival, were also similar between groups. Mechanical ventilation was operationally feasible in the prehospital setting despite several implementation challenges.

**Conclusion:**

In this exploratory prehospital pilot study, MV during CPR was feasible and produced preliminary physiologic measures comparable to BV. These findings are hypothesis-generating and may inform the design of future adequately powered studies.

**Trial registration:**

The study protocol was registered with the Thai Clinical Trials Registry (TCTR20241008007; 08 October 2024).

## Background

Out-of-hospital cardiac arrest (OHCA) remains a major global health challenge, with survival dependent on timely and effective resuscitation strategies [1–3]. Current guidelines emphasize high-quality cardiopulmonary resuscitation and early defibrillation, followed by advanced airway management and ventilation [4–6]. Ventilation during CPR is essential but must be carefully controlled, as both hypoventilation and hyperventilation may adversely affect outcomes [4, 7].

After advanced airway placement, ventilation can be delivered using either bag-valve ventilation (BV) or mechanical ventilation (MV) [8, 9]. BV is widely used due to its simplicity, but it may result in inconsistent ventilation, whereas MV allows more controlled delivery. However, ventilation during chest compressions is complex and may be influenced by changes in lung mechanics and intrathoracic pressure [9–14].

Previous studies have reported mixed findings regarding implementation and ventilation efficacy between MV and BV, particularly in in-hospital settings [15, 16]. In addition, recent evidence suggests that airway devices and ventilation strategies may significantly influence ventilation performance during CPR [17–19].

Therefore, this exploratory pilot study was primarily designed to evaluate the feasibility of implementing a standardized transport mechanical ventilation protocol during CPR within a physician-led EMS system and to generate preliminary physiologic data to inform the design of future definitive studies.

## Methods

### Trial design

This prospective, unblinded, time-sequenced exploratory pilot study was conducted within the Ramathibodi Emergency Medical Operation (EMO) unit, a physician-led emergency medical service (EMS) system based at Ramathibodi Hospital, Bangkok, Thailand. The EMO coordinates and deploys comprehensive life support (CLS) teams to provide advanced prehospital care for critically ill patients. These teams operate under the Bangkok EMS Center (Erawan Center) and are responsible for a defined service area (zone 8) surrounding the hospital.

Patients receiving BV were enrolled during the initial 6-month study phase, followed by patients receiving MV in the subsequent phase, which was originally planned for 6 months. BV had been the routine ventilation method prior to study initiation, consistent with common prehospital practice in Thailand and within the Ramathibodi EMO unit. This pragmatic time-sequenced approach was selected to facilitate real-world implementation of the ventilator protocol within the EMS system, as randomization was not feasible due to operational and training constraints.

Data collection was temporarily interrupted due to external operational disruptions affecting EMS deployment, resulting in an extension of the MV phase and overall study period until October 2025, without changes to the study protocol.

### Participants

Adult patients (≥ 18 years) with non-traumatic OHCA between September 2024 and October 2025 who required advanced airway management and ventilation during CPR were eligible for inclusion.

Exclusion criteria were return of spontaneous circulation (ROSC) prior to intubation or within 3 min after intubation; absence of arterial or venous blood gas measurements; traumatic cardiac arrest; suspected COVID-19 infection; suspected pneumothorax; pregnancy; do-not-attempt-resuscitation (DNAR) order; or lack of consent.

In the MV phase, cases were managed with BV instead of MV if it could not be applied due to operational constraints, such as confined scenes, equipment limitations, or device malfunctions.

### EMS team structure and protocol implementation

Each CLS team consisted of one emergency physician and paramedic personnel with predefined roles. The physician served as team leader and was responsible for clinical decision-making, rhythm assessment, and defibrillation. Paramedics were assigned to airway management, ventilation, drug administration, and operation of mechanical chest compression devices.

With the introduction of MV, team roles and workflows were further standardized to ensure a safe, coordinated implementation during ongoing CPR. The investigators developed a structured MV protocol in collaboration with the paramedic team. Prior to study implementation, all team members underwent focused training on airway management, ventilator operation, and role allocation during resuscitation, with particular emphasis on clear task allocation and team coordination. The initial ventilator settings were determined by the attending emergency physician or paramedic in accordance with predefined protocol. The protocol was disseminated to emergency physicians and paramedics through both direct training sessions and routine operational briefings.

### Airway management

All patients received endotracheal intubation as the advanced airway during resuscitation.

### Study intervention

In the MV phase, following intubation, patients were connected to a HAMILTON-T1 transport mechanical ventilator (Hamilton Medical, Switzerland) using volume-controlled ventilation mode. Depending on the clinical situation, ventilation was provided while the patient was resuscitated on scene, during preparation for transport, and throughout ambulance transport if resuscitation continued. The transport mechanical ventilation protocol was therefore intended to facilitate uninterrupted ventilation during patient transfer and ambulance transport rather than being restricted exclusively to the transport phase. Initial ventilator settings were tidal volume 6–8 mL/kg of ideal body weight, respiratory rate 10 breaths/min, PEEP 0 cmH₂O, inspiratory-to-expiratory (I: E) ratio 1:5, FiO₂ 1.0, peak pressure limit 60 cmH₂O, and flow trigger turned off.

These settings, including the use of zero PEEP, were selected based on local protocol and physiological considerations during CPR to minimise intrathoracic pressure and potential impairment of venous return during chest compressions.

In selected cases, PEEP could be adjusted (typically 0–5 cmH₂O) based on chest rise and clinical judgement.

### Control group

In the BV phase, following intubation, patients were ventilated using a self-inflating bag-valve device with a 1 L reservoir bag and oxygen flow of 15 L/min. Ventilation was delivered at a rate of approximately 10–12 breaths/min, guided by visible chest rise.

### Blood gas measurement

Blood gas analysis was performed using a point-of-care device (i-STAT Alinity, Abbott, USA). Sampling was conducted in the prehospital setting based on clinical feasibility. Arterial or venous samples were obtained depending on vascular access and patient condition.

Arterial and venous blood gases were analysed separately due to known physiological differences, particularly for PCO₂ in the setting of cardiac arrest. Blood samples were collected after at least 3 min of ventilation following intubation or upon ROSC, depending on clinical circumstances. Serial measurements were not feasible in the prehospital environment.

### Outcomes

The primary outcome of this pilot study was PCO₂ obtained from arterial or venous blood gas after at least 3 min of ventilation following intubation or upon ROSC, selected to provide a preliminary physiologic assessment of ventilation adequacy. Secondary outcomes included sustained ROSC (> 20 min) and 28-day survival, which were evaluated descriptively.

### Sample size calculation

As this was an exploratory pilot study, no formal sample size calculation was performed. The sample size was determined pragmatically based on case availability and operational feasibility. The study was not powered to detect statistically significant differences, and results are considered hypothesis-generating.

### Statistical methods

Stata version 17.0 (StataCorp LLC, College Station, TX, USA) was used for data analysis. Categorical variables were summarised as counts and percentages, and continuous variables as mean ± standard deviation (SD) or median (interquartile range [IQR]). The BV and MV groups were compared using the Chi-square or Fisher’s exact test for categorical data and the Student’s t-test or Mann–Whitney U test for continuous data.

Associations between ventilation strategy and physiologic or clinical outcomes were explored using univariable and multivariable regression analyses. PCO₂ was analysed using linear regression, and sustained ROSC was evaluated using logistic regression. Models were adjusted for selected potential confounders, and multicollinearity was assessed using the variance inflation factor. Given the exploratory pilot design and limited sample size, analyses were primarily descriptive, and regression estimates are presented with 95% confidence intervals to reflect the degree of uncertainty rather than to establish equivalence or superiority. Statistical significance was interpreted cautiously.

### Ethical considerations

The study was approved by the Human Research Ethics Committee of the Faculty of Medicine, Ramathibodi Hospital, Mahidol University (COA No. MURA2024/589; approved on 23 August 2024).

Patients received treatment according to emergency medical standards. Consent (from the patient’s immediate family members) was obtained at the time of (or after) study enrolment. If no family could be contacted, consent was obtained after the patient’s critical condition had stabilised or after contact was made with family members (deferred consent).

## Results

**Fig. 1:**
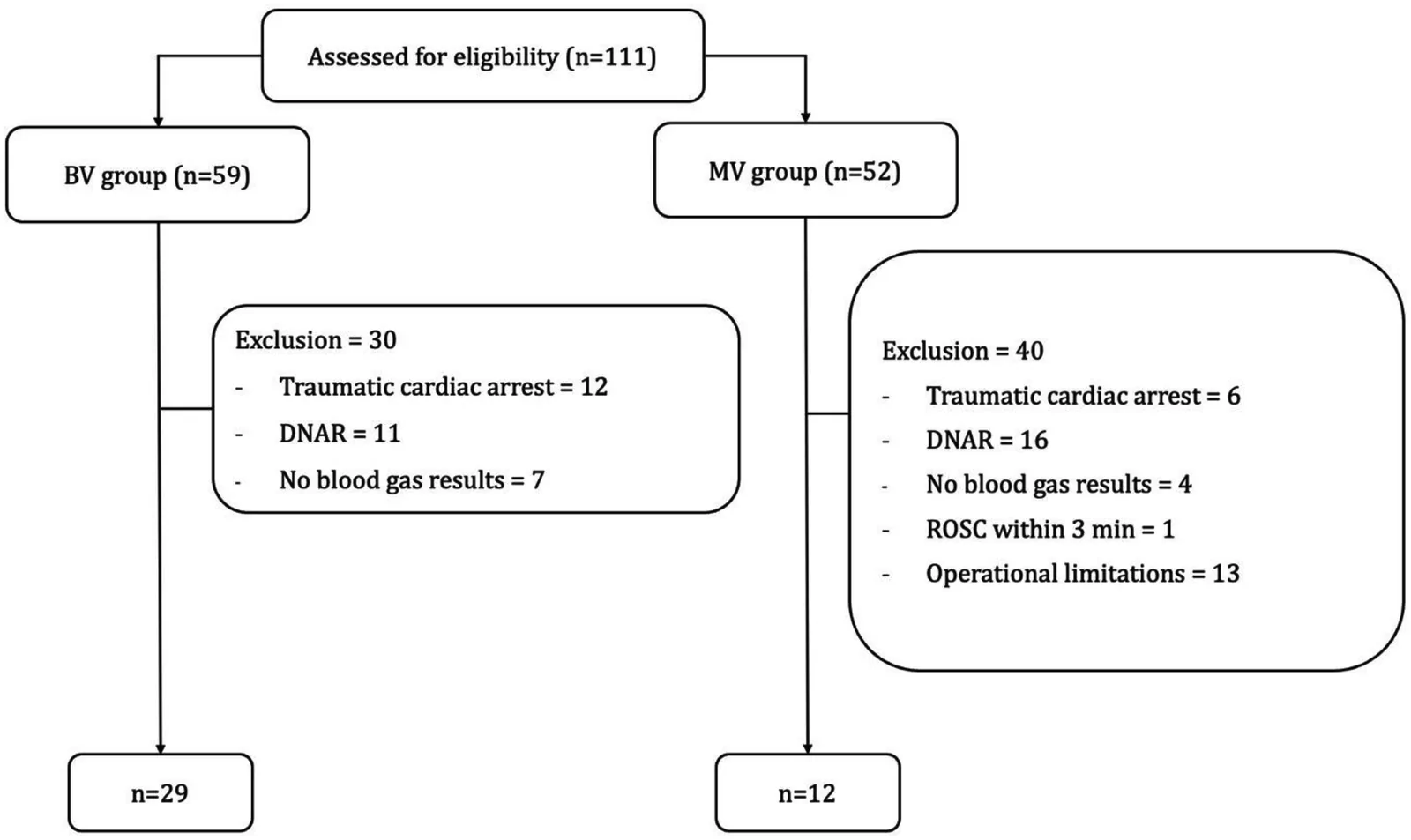
Participant flow

Overall, 111 patients met the eligibility criteria, and 70 were excluded (Fig. 1). In the MV phase, 13 eligible patients were managed with BV instead of MV because the transport ventilator could not be deployed under operational conditions. The most common implementation-related limitations were confined scene or transport-related constraints (*n* = 4), ventilator malfunction or monitoring system failure (*n* = 3), ventilator circuit disconnection or airflow leak alarms (*n* = 3), excessive airway secretion affecting ventilation (*n* = 2), and team readiness limitation during protocol implementation (*n* = 1). Ultimately, 41 patients were analysed (29 BV and 12 MV patients). There were no significant differences in baseline characteristics between the BV and MV groups (Table 1).

**Table 1:** Baseline characteristics and resuscitation outcomes of the BV group and the MV group

Variable	BV group (*n* = 29) *N*, %	MV group (*n* = 12) *N*, %
Male	16, 55.17	8, 66.67
Age mean ± SD	67.24 ± 15.21	65.58 ± 20.31
Diabetes mellitus	7, 24.14	3, 25.00
Hypertension	13, 44.83	2, 16.67
Dyslipidaemia	6, 20.69	1, 8.33
Previous stroke	1, 3.45	1, 8.33
Previous coronary artery disease	7, 24.14	2, 16.67
Obstructive airway disease	0, 0	1, 8.33
Chronic kidney disease	2, 6.90	0, 0
Cancer	0, 0	2, 16.67
Unknown underlying	5, 17.24	0, 0
Response time mean ± SD	16.52 ± 7.30	15.42 ± 5.03
Scene time mean ± SD	53.48 ± 13.67	47.50 ± 14.58
Collapse/detected cardiac arrest to CPR interval median (IQR)	20 (8, 34)	26.5 (17.5, 31)
Time of advanced airway placement (from collapse/detected cardiac arrest) median (IQR)	22 (12, 40)	33.5 (22, 35.5)
Time from intubation to blood gas median (IQR)	12 (9, 13)	12 (8.5, 13)
Time to first drug administration (from collapse/detected cardiac arrest) median (IQR)	27 (10, 40)	34 (22, 36)
Total CPR time median (IQR)	31 (23, 37)	29 (20, 34.5)
Shockable	2, 6.90	1, 8.33
Non-shockable	27, 93.10	11, 91.67
Cardiac	18, 62.07	5, 41.67
Hypoxia	19, 65.52	8, 66.67
Hypovolaemia	4, 13.79	1, 8.33
Hyperkalaemia	9, 31.03	5, 41.67
Acidosis	15, 51.72	8, 66.67
Total dose of adrenaline	7 (4, 8)	6 (4, 8)
Sustained ROSC	10, 34.48	7, 58.33
28-day survival	1, 3.45	0, 0

Abbreviations: BV, Bag-valve ventilation; MV, Mechanical ventilation; EMS, Emergency medical services; OHCA, out-of-hospital cardiac arrest; CPR, Cardiopulmonary resuscitation; ROSC, Return of spontaneous circulation

Arterial and venous blood gases were analysed separately. No statistically significant differences in ventilation-related parameters were detected between groups.

The median arterial PCO₂ was 69.7 mmHg (IQR 64.5–76.0) in the BV group and 81.9 mmHg (IQR 31.3–82.7) in the MV group. The mean venous PCO₂ was 86.76 ± 18.57 mmHg in the BV group and 97.43 ± 15.40 mmHg in the MV group (Table 2).

Regression analysis yielded an adjusted mean difference in PCO₂ of 1.04 mmHg (95% CI, − 13.91 to 16.00; *p* = 0.888) (Table 3).

Sustained ROSC and 28-day survival rates were numerically similar between groups (Table 1). The adjusted odds ratio for sustained ROSC was 3.17 (95% CI, 0.40–25.16; p = 0.274) (Table 4).

**Table 2:** Blood gas analysis and end-tidal CO_2_ of the BV group and the MV group

Variable	BV group (*n* = 29) *N*, %	MV group (*n* = 12) *N*, %
Arterial	*n* = 9	*n* = 5
pH	6.93 ± 0.13	6.94 ± 0.22
pO_2_	212 (95, 250)	84 (61, 102.3)
pCO_2_	69.7 (64.5, 76)	81.9 (31.3, 82.7)
HCO_3_^−^	15.56 ± 3.62	13.10 ± 3.70
Venous	*n* = 20	*n* = 7
pH	6.93 ± 0.11	6.84 ± 0.07
pO_2_	23 (13, 33)	22 (15, 32)
pCO_2_	86.76 ± 18.57	97.43 ± 15.40
HCO_3_^−^	18.89 ± 4.77	16.27 ± 3.99
End-tidal CO_2_	36.5 (20, 50)	43.5 (17, 68.5)

Abbreviations: BV, Bag-valve ventilation; MV, Mechanical ventilation

**Table 3:** Exploratory regression analysis of PCO₂ in the BV group and the MV group

Variable	Mean difference (95% CI)	*p*-value
PCO_2_ (unadjusted)	-0.49 (-16.28, 15.30)	0.950
PCO_2_ (adjusted*)	1.04 (-13.91,16.00)	0.888

*Adjusted for potential confounders (sex, age, arterial/venous, time of advanced airway placement, and time from intubation to blood gas measurement)

Abbreviations: BV, Bag-valve ventilation; MV, Mechanical ventilation

**Table 4:** Exploratory regression analysis of sustained ROSC in the BV group and the MV group

Variables	Odds ratio (95% CI)	*p*-value
Sustained ROSC (unadjusted)	2.66 (0.67, 10.57)	0.165
Sustained ROSC (adjusted*)	3.17 (0.40, 25.16)	0.274

*Adjusted for potential confounders (sex, age, response time, scene time, time to first drug administration, initial cardiac rhythm, pH, and end-tidal CO_2_)

Abbreviations: BV, Bag-valve ventilation; MV, Mechanical ventilation; ROSC, Return of spontaneous circulation

## Discussion

Rather than comparing clinical outcome, this study was intended to evaluate whether transport mechanical ventilation could be implemented safely and consistently within routine prehospital practice in our EMS system.

The MV and BV groups showed no statistically significant differences in blood gas parameters, including ventilation efficiency (as indicated by pCO₂), oxygenation, and acidosis. Similarly, sustained ROSC and 28-day survival were comparable.

These findings are consistent with a previous randomised controlled trial [16], which indicated no significant differences in gas exchange between MV and BV among patients with cardiac arrest presenting to the emergency department. In our prehospital setting, longer transport times may have prolonged the interval from cardiac arrest to CPR initiation and intubation, which may be associated with higher pCO₂ levels observed in this study. However, evidence from a systematic review and meta-analysis indicates no significant difference in hypercapnia versus normocapnia effects on mortality [20]. Another nonrandomized experimental study [21] that compared the efficacy of MV and BV in OHCA patients transported by EMS revealed adequate ventilation in both groups based on blood gas analysis upon emergency department arrival, with no significant difference. These differences could reflect variations in time from intubation to blood gas measurement (as influenced by the EMS system and transport distance) and missing blood gas data, suggesting possible selection and measurement bias in cardiac arrest studies.

In cardiac arrest, reduced pulmonary perfusion and apnoea cause alveolar collapse and atelectasis, which impair oxygenation. Arterial oxygen levels can decrease within 2 min, leading to hypoxia, hypercapnia and acidosis [22]. Chest compressions produce limited cardiac output and very small tidal volumes (< 1 mL/kg) that are insufficient for prolonged gas exchange [23]. Emerging ventilation strategies, such as chest-compression-synchronized ventilation (CCSV), have been developed to address these physiologic constraints. Experimental studies suggest that, despite delivering relatively low tidal volumes, CCSV may achieve more stable gas exchange during continuous chest compressions [18]. Therefore, appropriate assisted ventilation is required. Larger tidal volumes may improve hypercapnic acidosis and ventilation–perfusion mismatch, but the optimal ventilation rates remain uncertain [22]. Both hypoventilation and hyperventilation should be avoided according to current guidelines [4, 7].

MV provides more consistent minute ventilation by controlling respiratory rate and tidal volume. However, chest compressions reduce lung compliance and effective tidal delivery. A pilot randomised controlled trial [24] demonstrated lower tidal volume and minute ventilation with MV compared with BV, yet without differences in ROSC or blood gas outcomes, consistent with our findings. Ventilation performance is also influenced by airway device and delivery characteristics. Studies indicate that tracheal intubation provides more consistent tidal volumes and lower leakage than supraglottic airway devices [17, 19]. Thus, factors such as compliance, airway device, dead space, and pulmonary shunting may influence effective ventilation and explain the lack of observed differences between groups [22].

Conversely, another prospective observational study evaluated the efficacy of MV and BV in OHCA patients by utilising pCO₂ to measure ventilation [15]. The mean pCO₂ was significantly lower in the MV group than in the BV group, although no differences were observed in ROSC rates or survival to discharge. The proportion of patients with an initial shockable rhythm differed from that in our study, and the ventilator settings also differed, with a PEEP of 5 cmH₂O applied at initiation. A study on an animal model revealed that the application of PEEP during chest compressions improved minute ventilation, decreased pCO₂, prevented alveolar collapse, increased lung compliance and enhanced oxygenation [25]. However, the lack of comparable clinical studies in humans necessitates further research to determine optimal ventilation strategies that provide adequate gas exchange while maximising circulation during chest compressions in CPR.

In physician-led prehospital EMS systems, MV may reduce personnel burden; however, it requires coordinated teamwork and training. All patients in this study underwent endotracheal intubation, and alternative airway devices may influence ventilation performance and blood gas results, representing an area for future research.

Our study has several limitations. The small sample size, particularly in the MV group, limited the study’s statistical power. Similarly, because the study was conducted within a single EMS system, the findings may not be generalisable to other settings. The study was also unblinded, potentially introducing performance bias, and the time-sequenced design with imbalance between study phases may introduce selection bias. Most outcomes were based on short-term blood gas measurements, which may not fully reflect longer-term survival or neurological outcomes. In addition, blood gas samples were obtained either after at least 3 minutes following intubation or upon ROSC, representing distinct physiological states with different hemodynamic and gas exchange characteristics; this may introduce heterogeneity and limit direct comparability of measured parameters. Furthermore, arterial and venous blood gas measurements were used based on clinical feasibility and are not directly interchangeable, particularly for PCO₂, which may further limit interpretation of ventilation adequacy. Some samples were incomplete due to prehospital operational constraints, including difficulties transporting the mechanical ventilator to confined spaces and occasional equipment malfunctions. Additionally, detailed ventilation parameters after the initiation of BV or MV could not be collected due to personnel limitations, and ventilation pressures (including potential pressure-relief characteristics of bag-valve devices) were not directly measured, which may further limit interpretation of ventilation performance.

The exclusion of patients from the MV phase was primarily related to operational implementation challenges rather than patient characteristics. These exclusions should not be interpreted solely as equipment failures, but rather as implementation barriers encountered during early adoption of a new ventilation protocol in a real-world EMS system. Most excluded cases resulted from scene accessibility, equipment-related issues, or practical difficulties during deployment of the transport ventilator. These findings provide useful implementation information for EMS systems considering transport mechanical ventilation and identify important implementation barriers that should be addressed through improvements in equipment reliability, protocol refinement, and team training.

## Conclusion

In this exploratory prehospital pilot study, implementation of a standardized transport mechanical ventilation protocol during CPR was feasible within a physician-led EMS system. Although no significant differences were observed in physiologic or clinical outcomes, this pilot study was designed primarily to evaluate implementation feasibility rather than clinical effectiveness. These findings provide preliminary implementation and physiologic data to inform future adequately powered studies.

## Data Availability

The datasets generated and analysed during the current study are available from the corresponding author on reasonable request.
